# Effects of Dietary Luteolin on the Growth Performance and Intestinal Health of Juvenile GIFT Tilapia

**DOI:** 10.3390/antiox15080933

**Published:** 2026-07-28

**Authors:** Xiangli Li, Huige Ren, Jianting Lu, Yonggang Mo, Jingyi Du, Ye Qian, Xiao Peng, Zihe Guo, Chanxia Qin, Chengrui Huang, Kai Huang, Yinghui Zhang, Weihao Ou

**Affiliations:** 1 Key Laboratory of Aquatic Healthy Breeding and Nutrition Regulation of Guangxi Universities, College of Animal Science and Technology, Guangxi University, Nanning 530004, China; 2 Guangxi Xinshengtai Biotechnology Co., Ltd., Nanning 530004, China

**Keywords:** tilapia, luteolin, growth performance, antioxidant capacity, intestinal microbiota, intestinal metabolomics

## Abstract

This study aimed to investigate the effects of luteolin on the growth performance and intestinal health of juvenile Genetically Improved Farmed Tilapia (GIFT, *Oreochromis niloticus*). A total of 450 juvenile GIFT tilapia (initial body weight 8.12 ± 0.04 g) were randomly assigned to five groups (90 fish per group; three replicate tanks of 30 fish each) and fed diets containing luteolin at 0, 50, 150, 300, or 600 mg/kg (CG, LG50, LG150, LG300, and LG600, respectively) for 56 days. The results showed that with increasing luteolin supplementation, the weight gain rate (WGR) and specific growth rate (SGR) first increased and then decreased; the LG300 group had the highest WGR and SGR (significantly higher than the CG and LG600 groups, *p* < 0.05) and the lowest feed conversion ratio. Based on broken-line and quadratic polynomial regression analyses of the WGR and SGR, the optimal dietary luteolin supplementation level was determined to be 274.14–303.39 mg/kg. For intestinal antioxidant enzyme activities and oxidative stress, total superoxide dismutase (T-SOD) activity in the LG150 and LG300 groups was significantly higher than in the CG and LG600 groups (*p* < 0.05), and superoxide anion (O_2_^−^) content in the LG300 group was significantly lower than in the CG, LG50, and LG600 groups (*p* < 0.05). Histological analysis of the intestine revealed that muscular layer thickness was significantly greater in the LG300 group than in the CG group (*p* < 0.05). Intestinal microbiota analysis indicated that compared with the CG group, the LG300 group showed lower relative abundances of the potential pathogens *Plesiomonas* and *Bosea*, whereas it had a higher relative abundance of the potentially beneficial bacteria Bacteroidota and *Romboutsia*. Intestinal untargeted metabolomics revealed that, compared with the CG group, the beneficial metabolites Chrysoeriol, Laricitrin, and Docosahexaenoylethanolamine were significantly up-regulated, whereas the biotoxins Aplysiatoxin, 17-Debromo- and Pectenotoxin 3 were significantly down-regulated in the LG300 group (*p*-adjust < 0.05), with the up-regulated metabolites significantly enriched in the flavone and flavonol biosynthesis pathway (*p*-adjust < 0.05). Collectively, dietary supplementation with an appropriate level (300 mg/kg) of luteolin can effectively improve the growth performance and intestinal health of juvenile GIFT tilapia.

## 1. Introduction

The continuous growth of global aquatic product consumption has driven the rapid development of the aquaculture industry, which has become an important means of easing pressure on wild fishery resources. According to the Food and Agriculture Organization of the United Nations (FAO), global aquaculture production has increased by approximately 6.6% since 2020, reaching about 130.9 million tonnes and accounting for over 57% of aquatic animal products for direct human consumption [1,2]. In this context, tilapia has become the world’s second most farmed fish and is widely cultured in tropical and subtropical regions due to advantages such as its strong environmental adaptability, rapid growth, and low farming costs [2]. However, to maximize yields, the industry has widely adopted intensive, high-density farming models. Such systems inevitably subject fish to prolonged stress, which readily triggers intestinal dysfunction and consequently impairs growth performance and feed utilization [3,4,5]. Because the intestine governs nutrient digestion and absorption while serving as a central component of mucosal immunity, its structural and functional integrity is directly linked to fish growth and disease resistance, and any disruption ultimately undermines aquaculture profitability [6,7,8].

Intestinal health is commonly assessed using multi-dimensional indicators, including tissue structure, digestive enzyme activity, antioxidant capacity, and microbiota. Among these, antioxidant markers such as superoxide dismutase (SOD), catalase (CAT), and malondialdehyde (MDA) reflect the balance between the production and scavenging of reactive oxygen species, serving as sensitive indicators of intestinal oxidative status under stress. In addition, the advancement in omics, such as microbial high-throughput sequencing and metabolomics, can reveal host–microbiota interactions and identify key microbes, metabolites and pathways that link microbial shifts to host intestinal function [9]. These indicators have been widely used to evaluate feed additives in tilapia and other fish, providing a solid basis for related research [10,11,12,13].

Among feed additives, plant-derived natural bioactive substances—particularly flavonoids—have attracted wide interest in aquaculture for their safety, efficacy, and multi-target properties. Many studies have indicated that supplementing feed with appropriate flavonoids (such as quercetin, rutin, hesperidin, bamboo leaf flavonoids, and taxifolin) promoted aquaculture animals’ health and improved production efficiency while reducing reliance on antibiotics [14,15,16,17,18,19,20,21]. Luteolin (3′,4′,5,7-tetrahydroxyflavone) is a flavonoid compound widely found in various vegetables and fruits. Owing to its low toxicity and potent antioxidant, anti-inflammatory, and immunomodulatory activities, it has been extensively studied and applied in health foods and natural preservatives [22,23,24]. Recent studies have suggested considerable potential for luteolin in animal production. In early-weaned piglets, dietary luteolin improved daily weight gain and feed intake and alleviated soybean meal-induced intestinal damage by improving intestinal morphology and regulating the microbiota [25]. In red swamp crayfish, it increased survival and growth, enhanced hepatopancreatic antioxidant enzyme activity, and optimized the intestinal microbiota [26]. In largemouth bass, luteolin was the most effective of 15 tested compounds, inhibiting viral replication both in vitro and in vivo and raising the survival rate of infected fish [27]. To our knowledge, however, the effects of dietary luteolin on growth performance and intestinal health in fish—nor its appropriate dosage—have not been reported, particularly in tilapia.

Therefore, this study used Genetically Improved Farmed Tilapia (GIFT, *Oreochromis niloticus*) as the research subject. This improved strain is known for its fast growth and high meat yield, and is one of the major freshwater aquaculture species in China [28,29,30]. Benefits of luteolin in other animals are well established. Based on this, we hypothesized that dietary luteolin supplementation would improve the growth performance and intestinal health of juvenile GIFT. To test this, we systematically evaluated its effects on growth performance, intestinal antioxidant capacity, intestinal histological structure, intestinal microbiota, and intestinal metabolic profiles, aiming to provide a theoretical basis and practical reference for the scientific application of luteolin in aquaculture.

## 2. Materials and Methods

### 2.1. Experimental Design and Feed Preparation

Luteolin (purity ≥ 98%) was purchased from Chengdu Push Bio-technology Co., Ltd. (Chengdu, China). The composition and formulation of the basal diet for juvenile GIFT tilapia are presented in Table 1 [31]. Luteolin was supplemented into the basal diet at levels of 0 (CG, the control group), 50 (LG50 group), 150 (LG150 group), 300 (LG300 group), and 600 (LG600 group) mg/kg. The preparation of the experimental diets followed the methods described by Zhang et al. [32] and Othman et al. [33]. Briefly, luteolin was dissolved in 95% ethanol and serially diluted to the target concentrations. The solutions were evenly sprayed onto the surface of the feed powder and thoroughly mixed to prepare the respective diets. The control group (CG) was sprayed with an equal volume of 95% ethanol solution without luteolin. All diets were air-dried in a ventilated area, packed into sealed bags, and stored at −20 °C. The final ethanol residue in the feed was less than 0.1%, which is well below the reported No-Observed-Effect Concentration (NOEC) for fish [34]. Therefore, we confirmed that the final ethanol concentration was extremely low and unlikely to interfere with fish physiological activities. The addition level of luteolin in the feed was primarily determined based on the existing research reports [25,26,35].

### 2.2. Experimental Fish and Feeding Management

The juvenile GIFT tilapia used in this study were sourced from an aquaculture farm in Nanning, Guangxi, China. Upon arrival, the fish were acclimated for 2 weeks while being fed the basal diet. After acclimation, a total of 450 healthy fish with uniform size and an initial body weight of 8.12 ± 0.04 g were selected and randomly divided into 5 groups (3 tanks per group, each tank was 400 L and stocked with 30 fish) according to the experimental design. Each group was fed its corresponding diet. The feeding trial lasted for 56 days. The fish were fed to apparent satiation twice daily at 8:00 and 16:00. Uneaten feed and feces were siphoned out promptly after each feeding. Approximately 30% of the water in each tank was exchanged daily. During the rearing period, water quality parameters were maintained within the following ranges: temperature 26–30 °C, pH 6.5–7.2, dissolved oxygen > 5.0 mg/L, ammonia nitrogen < 0.3 mg/L, and nitrite < 0.1 mg/L. Fish behavior and health status were observed daily, and mortality as well as feed consumption were recorded.

### 2.3. Sample Collection

At the end of the feeding trial, all fish were fasted for 24 h. Subsequently, they were anesthetized with an appropriate amount of eugenol, counted, and weighed. Using sterilized dissecting instruments and operating near the flame of an alcohol burner, the hindguts were collected after being rinsed with physiological saline. The hindguts of 2 fish randomly selected from each tank were pooled into one pooled sample, placed into enzyme-free and sterile 2 mL centrifuge tubes, immediately snap-frozen in liquid nitrogen, and finally stored in a −80 °C freezer. Finally, 3 pooled samples per group were obtained for intestinal enzyme activity analysis, 6 for intestinal microbiota analysis, and 6 for intestinal untargeted metabolomics analysis. Additionally, one fish was randomly selected from each tank, and its hindgut was collected and placed into a 5 mL centrifuge tube containing 4% paraformaldehyde for histological analysis.

### 2.4. Growth Performance Analysis

The following formula served as a basis for calculating growth performance: W_i_ (g) represents the initial body weight, W_f_ (g) represents the final body weight, t (days) represents the number of feeding days, and FI (g) represents the amount of feed provided.

$$\mathrm{Weight}\;\mathrm{Gain}\;\mathrm{Rate}\;(WGR,\%)=100\times \left(W_{f}-W_{i}\right)/W_{i}$$

$$\mathrm{Specific}\;\mathrm{Growth}\;\mathrm{Rate}\;(SGR,\%/d)=100\times \left(\ln W_{f}-\ln W_{i}\right)/t$$

$$\mathrm{Feeding}\;\mathrm{Rate}\;(\mathrm{FR},\%/d)=100\;\times \;\mathrm{FI}/((W_{i}+W_{f})/2)\times t$$

Feed Conversion Ratio (FCR) = FI/(W_f_ − W_i_)

### 2.5. Intestinal Antioxidant Enzyme Activities and Oxidative Stress Assays

After thawing, the collected intestinal tissue samples were mixed with pre-cooled phosphate-buffered saline (PBS, 0.01 mol/L, pH 7.4) at a ratio of 1:9 (*w*/*v*), and the mixture was thoroughly homogenized in an ice-water bath. Subsequently, the homogenate was centrifuged at 560× *g* for 10 min at 4 °C, and the supernatant was collected for analysis. The activities of the intestinal antioxidant enzymes catalase (CAT; Cat. No. A007-1-1) and total superoxide dismutase (T-SOD; Cat. No. A001-3-2), together with the levels of the oxidative stress marker malondialdehyde (MDA; Cat. No. A003-1-2) were determined using the corresponding assay kits from Nanjing Jiancheng Bioengineering Institute (Nanjing, China). In addition, the levels of another oxidative stress marker superoxide anion (O_2_^−^; Cat. No. BC1295) were determined using the corresponding assay kits from Beijing Solarbio Science & Technology Co., Ltd. (Beijing, China).

### 2.6. Intestinal Histological Evaluation

The intestinal samples were fixed in 4% paraformaldehyde fixative (Servicebio, Wuhan, China) at 4 °C for 24 h. The fixed samples were then trimmed, dehydrated through a graded ethanol series, cleared in xylene, and embedded in paraffin to prepare sections. The sections were then subjected to Hematoxylin and Eosin (H&E) staining and mounting. Tissue morphology was observed under a Biological Microscope BX53F2 (Olympus Corporation, Tokyo, Japan), and images were captured using cellSens Standard software (version 1.18; Olympus Corporation, Tokyo, Japan). Finally, the captured images were analyzed using ImageJ software (Version 1.54p; National Institutes of Health, Bethesda, MD, USA) to measure intestinal morphological parameters. Following the method described by [36], three sections were randomly selected from each group. For each section, ten well-defined, complete, and nearly vertically oriented mucosal folds were chosen to measure their fold height (the vertical distance from the tip to the base of the fold). The total number of intact and independent intestinal folds within each section was counted (partially or completely fused folds were counted as one). Additionally, the thickness of muscular layer was measured at ten locations within the intestine corresponding to the selected folds. The data obtained from each group were averaged for subsequent statistical analysis. In addition, all sections were qualitatively examined for epithelial integrity (continuity and arrangement of the mucosal epithelium) and inflammatory cell infiltration.

### 2.7. Intestinal Microbiota Testing

Total genomic DNA of intestinal microbiota was extracted using the E.Z.N.A.^®^ soil DNA Kit (Omega Bio-tek, Norcross, GA, USA), and its quality and concentration were assessed by 1% agarose gel electrophoresis and a NanoDrop 2000 spectrophotometer (Thermo Scientific, Wilmington, DE, USA). Using the extracted genomic DNA as a template, the V3-V4 variable regions of the 16S rRNA gene were selected for PCR amplification. The primers used were 338F (5′-ACTCCTACGGGAGGCAGCAG-3′) and 806R (5′-GGACTACHVGGGTWTCTAAT-3′) [37]. The PCR reaction was performed on a T100 Thermal Cycler (Bio-Rad Laboratories, Hercules, CA, USA). PCR amplification adopted a 20 μL reaction system, including 10 μL of 2 × Pro Taq, 0.8 μL each of forward and reverse primers (5 μM), and 10 ng of template DNA, with the remaining volume made up to 20 μL with ddH_2_O. The amplification program was as follows: pre-denaturation at 95 °C for 3 min; 29 cycles (denaturation at 95 °C for 30 s, annealing at 53 °C for 30 s, and extension at 72 °C for 45 s); and a final extension at 72 °C for 10 min. The amplification products were purified by recovery from 2% agarose gel electrophoresis and quantified using Qubit 4.0 Fluorometer (Thermo Fisher Scientific, Waltham, MA, USA). 

The purified amplification products were used to construct sequencing libraries using the NEXTFLEX^®^ Rapid DNA-Seq Kit (Bioo Scientific, Austin, TX, USA). Paired-end sequencing was performed using the Illumina PE300 platform (Illumina, San Diego, CA, USA). The data for each sample were demultiplexed from the raw data based on Barcode sequences and PCR amplification primer sequences. Raw reads were quality-controlled using fastp [38] (Version 0.23.4; https://github.com/OpenGene/fastp (accessed on 10 May 2025)), and sequence merging was performed using FLASH [39] (Version 1.2.11; http://ccb.jhu.edu/software/FLASH (accessed on 10 May 2025)). Denoising was processed via the DADA2 plugin in QIIME2 [40] (https://qiime2.org (accessed on 10 May 2025)) to generate Amplicon Sequence Variants (ASVs). All sample sequences were rarefied based on the minimum sample sequence count. Taxonomic annotation of ASVs was performed based on the Silva 16S rRNA gene database (Version 138; https://www.arb-silva.de/ (accessed on 10 May 2025)), and chloroplast and mitochondria sequences were removed from all samples. Mothur [41] (Version 1.48.3) was used to calculate Alpha diversity indices, including Chao1, Observed-richness, Shannon, and Simpson indices, and the Wilcoxon rank-sum test was employed for comparisons between two groups. Using R (Version 4.2.0; R Foundation for Statistical Computing, Vienna, Austria), Principal coordinate analysis (PCoA) based on abund_jaccard distance was performed and plotted, combined with PERMANOVA non-parametric test to evaluate differences in community structure between groups; LEfSe analysis (Linear discriminant analysis Effect Size) [42] (http://huttenhower.sph.harvard.edu/LEfSe (accessed on 11 May 2025)) (LDA > 2, *p* < 0.05) was used to identify bacteria with significantly different abundances from phylum to genus levels between different groups. The raw data of 16S rRNA gene amplicon sequencing had been uploaded to the Sequence Read Archive (SRA) with the accession number PRJNA1445903.

### 2.8. Intestinal Untargeted Metabolomics Assays

Untargeted metabolomics analysis was performed using Liquid Chromatography-Tandem Mass Spectrometry (LC-MS/MS). First, 100 ± 5 mg of intestinal samples were accurately weighed, and 400 µL of pre-cooled extraction solution [methanol:water, 4:1 (*v*/*v*), containing 0.02 mg/mL L-2-chlorophenylalanine (Sigma-Aldrich, St. Louis, MO, USA) as the internal standard] was added. The samples were ground at low temperature (−10 °C, 50 Hz) for 6 min using a Wonbio-96c frozen tissue grinder (Shanghai Wanbo Biotechnology Co., Ltd., Shanghai, China), followed by low-temperature ultrasonic extraction (5 °C, 40 kHz) for 30 min using an ultrasonic bath (SBL-10DT ultrasonic cleaner 300W-10L, Ningbo Scientz Biotechnology Co., Ltd., Ningbo, China). After standing at −20 °C for 30 min, the mixture was centrifuged at 13,000× *g* for 15 min at 4 °C using a refrigerated centrifuge (Centrifuge 5430R high-speed refrigerated centrifuge, Eppendorf AG, Hamburg, Germany), and the supernatant was collected for analysis. To monitor instrument status and the stability of the analytical process, 20 µL aliquots from each sample supernatant were pooled to prepare Quality Control (QC) samples. Chromatographic separation was performed on a UHPLC-Exploris240 system (Thermo Fisher Scientific, Waltham, MA, USA) using an ACQUITY UPLC HSS T3 column (100 mm × 2.1 mm i.d., 1.8 µm; Waters, Milford, MA, USA). Mobile phase A consisted of 95% water + 5% acetonitrile (containing 0.1% formic acid), and mobile phase B consisted of 47.5% acetonitrile + 47.5% isopropanol + 5% water (containing 0.1% formic acid). The injection volume was set to 3 µL, and the column temperature was maintained at 40 °C. Mass spectrometry data acquisition was performed in both positive and negative ion scanning modes, with a scan range of *m*/*z* 70–1050 to ensure broad coverage of the metabolic profile.

The raw data were imported into Progenesis QI v3.0 (Waters Corporation, Milford, MA, USA) for baseline filtering, peak identification, integration, retention time correction, and peak alignment, generating a data matrix containing information such as retention time, mass-to-charge ratio (*m*/*z*), and peak intensity. Feature peaks were identified based on public databases such as HMDB (http://www.hmdb.ca/ (accessed on 10 May 2025)) and METLIN (https://metlin-nl.scripps.edu/ (accessed on 10 May 2025)). The mass error threshold for MS1 was set at 10 ppm, and metabolite identification was performed by combining MS2 spectral matching scores. Subsequent analysis was conducted on the Majorbio Cloud Platform (https://www.majorbio.com/ (accessed on 10 May 2025)). To eliminate or minimize errors introduced during the experimental and analytical processes, the qualitative data underwent preprocessing. This included removing features where more than 20% of the values were missing within any single group, then filling the remaining missing values with the minimum value from all samples. The peak intensity for each sample was normalized using the sum normalization method, resulting in a normalized data matrix. Variables with a Relative Standard Deviation (RSD) > 30% in the QC samples were removed, followed by log10 transformation, yielding the final data matrix for subsequent analysis.

Partial Least Squares Discriminant Analysis (PLS-DA) and Orthogonal Partial Least Squares Discriminant Analysis (OPLS-DA) were performed using the ropls package (version 1.6.2) in R. Metabolite pathways were annotated against the HMDB and KEGG (https://www.kegg.jp/kegg/pathway.html (accessed on 17 May 2025)) databases, and pathway enrichment analysis was performed using the scipy.stats package (version 1.0.0) in Python. To control the false discovery rate, *p*-values were adjusted using the Benjamini–Hochberg method (*p*-adjust). Metabolites satisfying VIP > 1.5 (from the OPLS-DA model) and *p*-adjust < 0.05 were considered significantly different between groups, with the fold change (FC, the ratio of mean relative abundance, LG300/CG) indicating the direction of regulation (FC > 1, up-regulated; FC < 1, down-regulated). The top 20 up- and down-regulated metabolites ranked by *p*-adjust were further selected.

### 2.9. Statistical Analysis

All data are expressed as mean ± SD. Except for the microbiota and untargeted metabolomics data, all data were analyzed using SPSS (Version 26.0; IBM Corporation, Armonk, NY, USA), and figures were plotted with GraphPad Prism (Version 9.0; GraphPad Software, San Diego, CA, USA). Normality and homogeneity of variance were assessed using the Shapiro–Wilk and Levene’s tests, respectively. Differences among three or more groups were analyzed by one-way ANOVA followed by Duncan’s (equal variances) or Games-Howell (unequal variances) post hoc test, whereas comparisons between two groups were performed using the independent-samples *t*-test. As the microbiota and untargeted metabolomics data were not normally distributed, two-group comparisons for these datasets were conducted using the Wilcoxon rank-sum test (Mann–Whitney U test) within their respective analysis pipelines. The optimal luteolin levels for WGR and SGR were estimated by piecewise linear and quadratic polynomial regression in R. Differences were considered statistically significant at *p* < 0.05.

## 3. Results

### 3.1. Growth Performance

**Figure 1:**
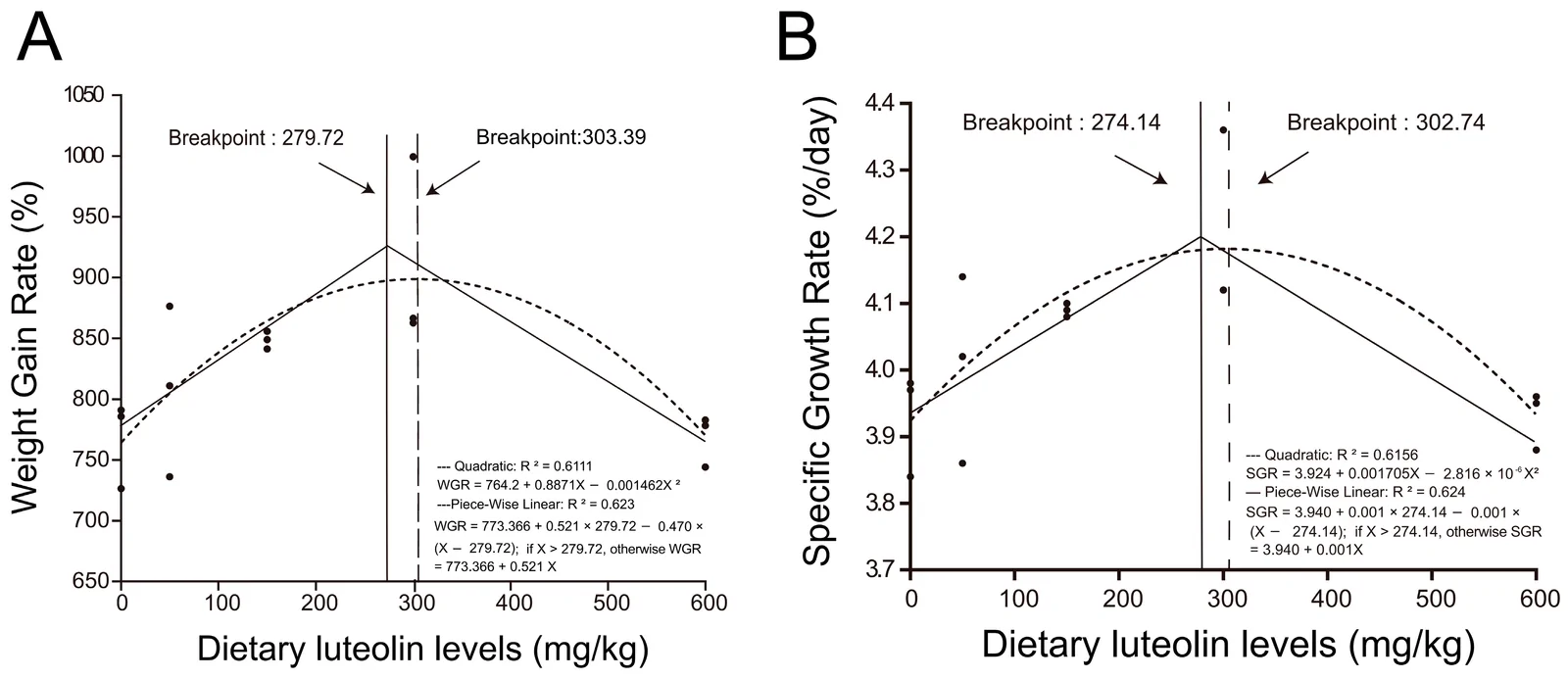
Dose–response analysis of weight gain rate (**A**) and specific growth rate (**B**) to dietary luteolin levels for estimating the optimal requirement. Notes: *n* = 3 per group, total *n* = 15; each dot represents an individual biological replicate. The solid line represents the piecewise linear regression model and the dashed line represents the quadratic polynomial regression model; arrows indicate the break-point of the piecewise linear model and the vertex of the quadratic curve, respectively, corresponding to the estimated optimal dietary luteolin supplementation level.

Table 2 shows the effects of dietary supplementation with different levels of luteolin on the growth performance of juvenile GIFT tilapia. With the increase in luteolin supplementation, WGR and SGR increased first and then decreased, with the LG300 group being significantly higher than the CG and LG600 groups (*p* < 0.05). There were no significant differences in FBW, FR, and FCR among the groups (*p* > 0.05). To determine the optimal supplementation level of luteolin, regression models were fitted for WGR and SGR. The curves and their equations are presented in Figure 1. The results of piecewise linear analysis indicated that the optimal luteolin supplementation levels determined based on WGR and SGR were 279.72 mg/kg and 274.14 mg/kg, respectively. Whereas the optimal luteolin supplementation levels determined according to quadratic regression analysis were 303.39 mg/kg and 302.74 mg/kg, respectively.

### 3.2. Intestinal Antioxidant Enzyme Activities and Oxidative Stress

**Figure 2:**
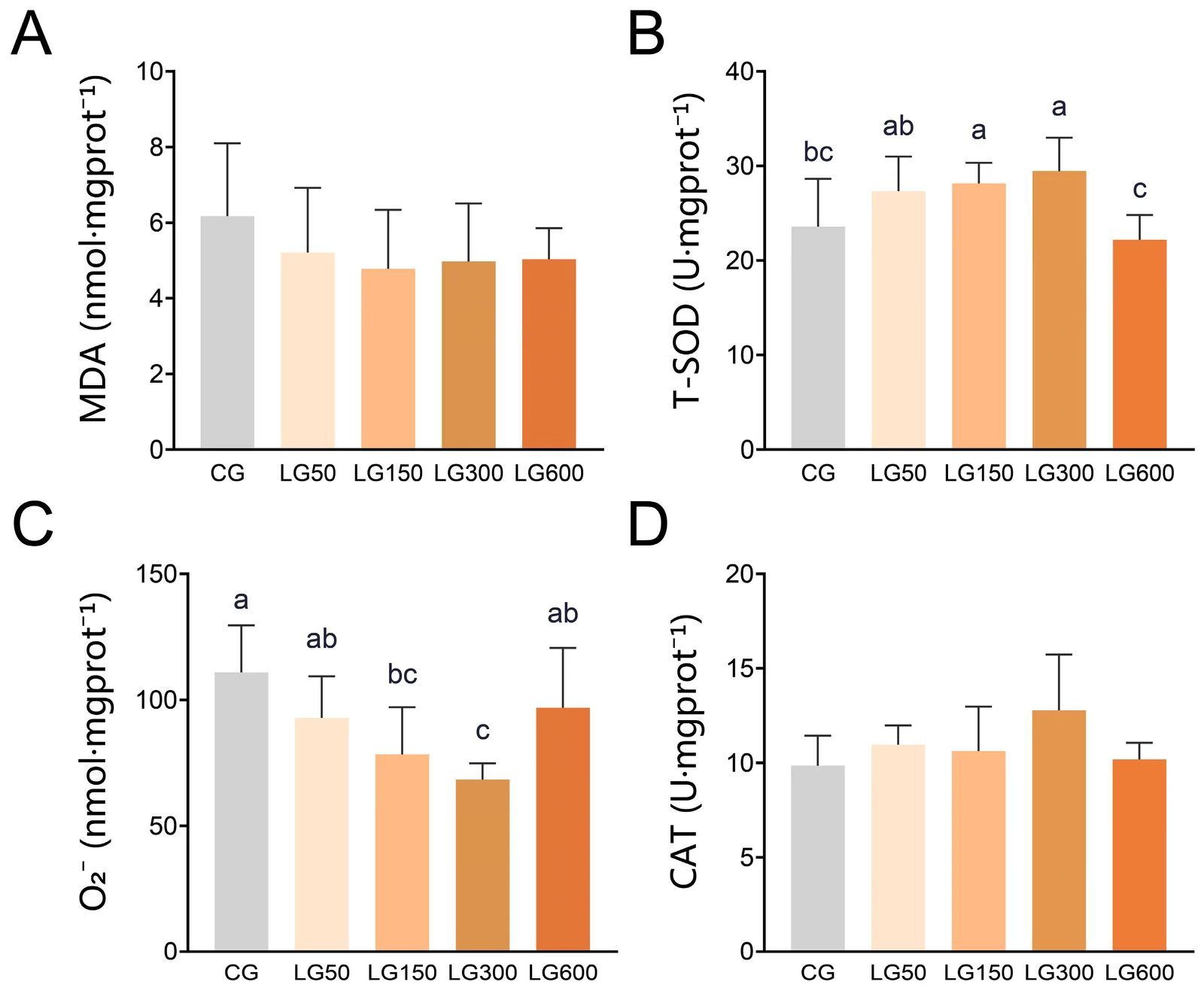
Effects of luteolin-supplemented diet on the intestinal antioxidant enzyme activity and oxidative stress of juvenile GIFT tilapia. Notes: Data are presented as mean ± SD (*n* = 3 per group, total *n* = 15). (**A**) malondialdehyde (MDA) content; (**B**) total superoxide dismutase (T-SOD) activity; (**C**) superoxide anion (O_2_^−^) content; (**D**) catalase (CAT) activity. Within each panel, bars that do not share same letter are significantly different, whereas bars sharing same letter or with no letter are not significantly different (one-way ANOVA followed by post hoc test; *p* < 0.05 indicates significant difference). CG: control group; LG50, LG150, LG300 and LG600: groups supplemented with 50, 150, 300 and 600 mg/kg luteolin in the basal diet, respectively.

The intestinal antioxidant enzyme activities and oxidative stress markers of juvenile GIFT tilapia are shown in Figure 2. The T-SOD activity in the LG150 and LG300 groups was significantly higher than that in the CG and LG600 groups (*p* < 0.05). The O_2_^−^ content in the LG300 group was significantly lower than that in the CG, LG50, and LG600 groups (*p* < 0.05). In addition, the luteolin-supplemented groups showed an increasing trend in intestinal CAT activity and a decreasing trend in MDA content, although no significant differences were observed among the groups for these two parameters (*p* > 0.05).

### 3.3. Histology Analyses of the Intestine

**Figure 3:**
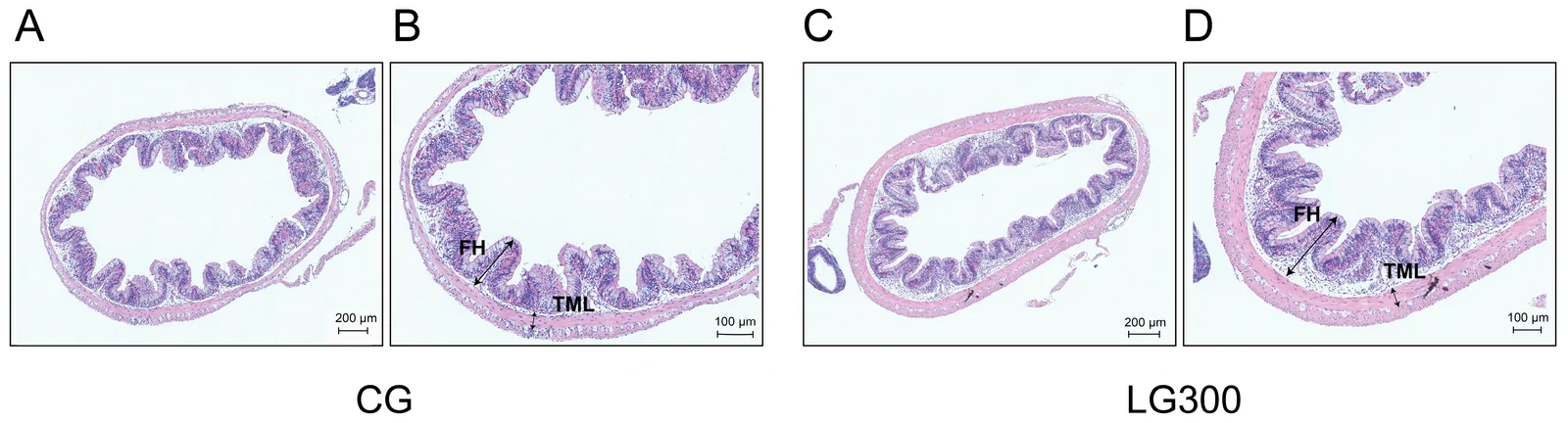
Intestinal histology of juvenile GIFT tilapia from the CG and LG300 groups. Notes: *n* = 3 per group, total *n* = 6. (**A**,**B**) CG (control group); (**C**,**D**) LG300 (group supplemented with 300 mg/kg luteolin in the basal diet). (**A**,**C**) H&E staining, 80×; (**B**,**D**) H&E staining, 160×. FH: fold height; TML: thickness of muscular layer.

Based on the data in Figure 1 and Figure 2, we analyzed the intestinal histomorphology of the CG and the LG300 group, which achieved the best growth performance and intestinal antioxidant capacity, as shown in Figure 3. The results indicated that compared with the control group, the LG300 group showed no significant changes in fold height or fold number (*p* > 0.05) (Table 3). However, the thickness of the muscular layer in the LG300 group was significantly higher than that in the CG group (*p* < 0.05) (Table 3). In both groups, the mucosal epithelium remained intact with closely and regularly arranged epithelial cells, and no obvious inflammatory cell infiltration or epithelial detachment was observed (Figure 3).

### 3.4. Intestinal Microbiota

**Figure 4:**
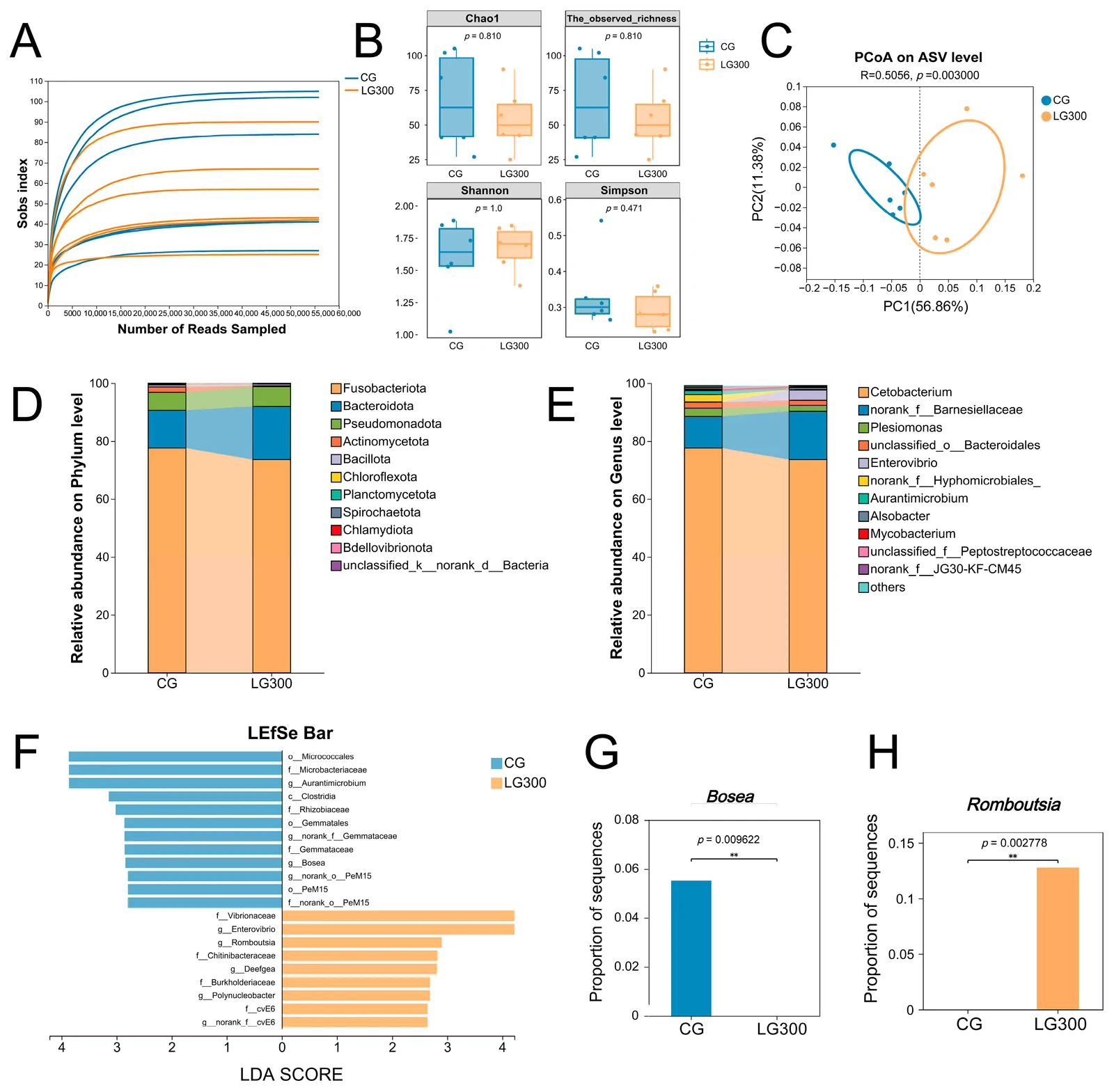
Intestinal microbiota analysis of juvenile GIFT tilapia from the CG and LG300 groups. Notes: *n* = 6 per group, total *n* = 12. (**A**) Rarefaction curve; (**B**) alpha diversity indices (Chao1, Observed richness, Shannon, and Simpson); (**C**) principal coordinate analysis (PCoA) plot based on the abund_jaccard distance; (**D**) microbiota composition at the phylum level; (**E**) microbiota composition at the genus level; (**F**) linear discriminant analysis effect size (LEfSe) analysis (linear discriminant analysis [LDA] score > 2, *p* < 0.05); (**G**) relative abundance of *Bosea*; (**H**) relative abundance of *Romboutsia*. Alpha diversity indices and the relative abundances in (**G**,**H**) were compared between the two groups using the Wilcoxon rank-sum test, and the community structure in (**C**) was tested by PERMANOVA. *p* < 0.05 indicates significant difference. Asterisks indicate significant differences between groups (** *p* < 0.01). CG: control group; LG300: group supplemented with 300 mg/kg luteolin in the basal diet.

To elucidate how dietary luteolin at its optimal effective dose reshapes the intestinal microbial community, we further analyzed the intestinal microbiota of the CG and LG300 groups, as the LG300 group exhibited the best growth performance and intestinal antioxidant enzyme activities among all luteolin-supplemented groups. As shown in Figure 4A, with the increase in sequencing depth, the number of newly discovered species in all samples gradually approached saturation, and the rarefaction curves ultimately plateaued. This indicated that the current sequencing depth was sufficient to comprehensively reflect the characteristics of the intestinal microbiota in each sample. Analysis of the Alpha diversity indices (Figure 4B) of the intestinal microbiota in juvenile GIFT tilapia showed no significant differences between the CG and LG300 groups in terms of species richness (Chao1 and Observed species indices) or community diversity (Shannon and Simpson indices) (*p* > 0.05).

At the ASVs level of the intestinal microbiota, PCoA was performed using the abund_jaccard distance algorithm. As shown in Figure 4C, samples from the CG and LG300 groups formed distinct, separate clusters on the plot. The Adonis test further confirmed that there were significant differences in the intestinal microbiota structure between the two groups (*p* < 0.01), indicating that luteolin could significantly alter the overall composition of the intestinal microbiota. Figure 4D shows that the top three dominant phyla in the intestine of juvenile GIFT tilapia were Fusobacteriota, Bacteroidota, and Pseudomonadota. Moreover, compared with the CG group, the relative abundance of Fusobacteriota in the LG300 group decreased, while the relative abundance of Bacteroidota and Pseudomonadota increased. At the genus level (Figure 4E), the top three dominant genera were *Cetobacterium*, norank_f__Barnesiellaceae, and *Plesiomonas*. Compared with the CG group, the relative abundances of *Cetobacterium* and *Plesiomonas* decreased in the LG300 group, while the relative abundance of norank_f__Barnesiellaceae increased.

Based on the screening criteria of LDA score > 2.0 and *p* < 0.05, a bar chart of the LDA values of LEfSe was plotted (Figure 4F). The results showed that in the CG group, the significantly enriched microbial taxa included: Clostridia at the class level; Micrococcales, Gemmatales, and PeM15 at the order level; Microbacteriaceae, Rhizobiaceae, Gemmataceae, and f__norank_o__PeM15 at the family level; and *Aurantimicrobium*, *Bosea*, g__norank_f__Gemmataceae, and g__norank_o__PeM15 at the genus level. However, in the LG300 group, significantly enriched microbes included: Vibrionaceae, Chitinibacteraceae, Burkholderiaceae, and f__cvE6 at the family level; and *Enterovibrio*, *Romboutsia*, *Deefgea*, *Polynucleobacter* and g__norank_f__cvE6 at the genus level.

Additionally, the relative abundance of *Bosea* in the CG group was significantly higher than that in the LG300 group, and it was not detected in the LG300 group (Figure 4G). The relative abundance of *Romboutsia* was significantly higher in the LG300 group than in the CG group, and it was not detected in the CG group (Figure 4H).

### 3.5. Intestinal Untargeted Metabolomics Analysis

To deeply explore the mechanism by which luteolin affected the intestinal health of juvenile GIFT tilapia from the perspective of host metabolism, we employed untargeted metabolomics to analyze the intestinal metabolites of the CG and LG300 groups. In metabolite identification, 1923 and 2427 metabolites were obtained through positive and negative ion mode detection, respectively (Table 4). For subsequent analyses, the datasets acquired under positive and negative ion modes were merged to maximize metabolite coverage. Database matching against HMDB and KEGG revealed that, within this combined dataset, the metabolites identified and annotated from the negative ion mode outnumbered those from the positive ion mode, making it the major contributor to the feature pool used for the screening of differential metabolites between groups (Table 4). It is important to note that the two ionization modes were complementary; the broader coverage for comprehensive metabolomic profiling was achieved through their integration, and the higher abundance of annotations in the negative mode merely reflected the physicochemical properties of the metabolites present in our specific sample matrix.

**Figure 5:**
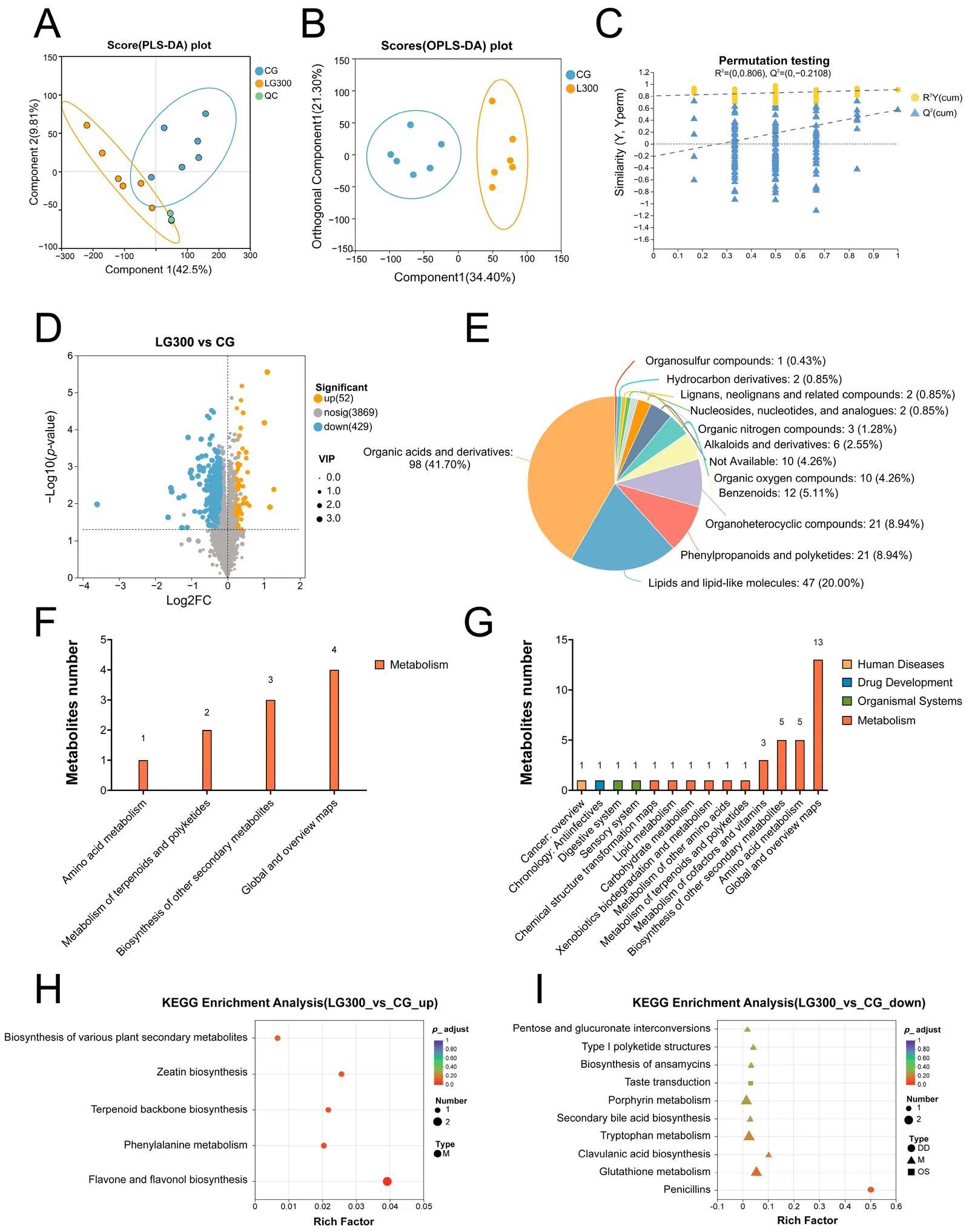
Intestinal untargeted metabolomics analysis of juvenile GIFT tilapia from the CG and LG300 groups. Notes: *n* = 6 per group, total *n* = 12. (**A**) Partial least squares discriminant analysis (PLS-DA) score plot; (**B**) orthogonal partial least squares discriminant analysis (OPLS-DA) score plot; (**C**) permutation test of the OPLS-DA model; (**D**) volcano plot of significantly differential metabolites; (**E**) classification of significantly differential metabolites based on the HMDB database; (**F**) KEGG level 2 pathway annotation of significantly up-regulated metabolites; (**G**) KEGG level 2 pathway annotation of significantly down-regulated metabolites; (**H**) KEGG level 3 pathway enrichment of significantly up-regulated metabolites; (**I**) KEGG level 3 pathway enrichment of significantly down-regulated metabolites. *p*-adjust: Benjamini–Hochberg (BH) adjusted *p*-value; *p*-adjust < 0.05 indicates significant difference. CG: control group; LG300: group supplemented with 300 mg/kg luteolin in the basal diet.

The results of PLS-DA (Figure 5A) and OPLS-DA (Figure 5B) showed distinct clustering of samples within each group, a high degree of separation of metabolites between the two groups, and the absence of any outlier samples. These findings indicated that luteolin intervention significantly altered the overall intestinal metabolic profile. To assess the quality of the model, we performed a permutation test on the OPLS-DA model. The results, R^2^ = (0, 0.806) and Q^2^ = (0, −0.2108), demonstrated that the model was robust and reliable, with no overfitting, and possessed good repeatability and predictive capability (Figure 5C). To identify key differential metabolites, metabolites were required to simultaneously satisfy VIP > 1.5 and *p*-adjust < 0.05, with FC (LG300/CG) indicating the direction of regulation. Compared with the CG group, 52 metabolites were upregulated and 429 were downregulated in the LG300 group (Figure 5D). Classification of all differential metabolites by HMDB showed that Organic acids and derivatives, Lipids and lipid-like molecules, and Organoheterocyclic compounds were the top three categories with the highest proportions of differential metabolites (Figure 5E).

Table 5 presented the differential metabolites that were significantly up-regulated or down-regulated in the LG300 group compared with the CG group (top 20 listed). The significantly up-regulated differential metabolites were: 5′-(3′,4′-Dihydroxyphenyl)-Gamma-Valerolactone Sulfate, Chrysoeriol, Propranolol Glucuronide, Diosmetin, Meosuc-Aapa-Cmk, 4-Hydroxy-5-(Phenyl)-Valeric Acid-O-Sulphate, Quinoneimine, Selpercatinib, Laricitrin, 2-C-Methyl-D-Erythritol 2,4-Cyclodiphosphate, Docosahexaenoylethanolamine, Kaempferol 3-Glucuronoside, Pe(5-Iso Pgf2Vi/22:6(4Z,7Z,10Z,13Z,16Z,19Z)), 3-Methyladipic Acid, Glu-Phe-Tyr, Elarofiban, Glu-Ile-Phe-Lys, Cyclo[Dl-Pro-Dl-Pro-Dl-Val-Dl-Tyr-Dl-Tyr], Trinexapac, and Ginsenoside A2. The significantly down-regulated differential metabolites included: Gly-Glu-Arg, Aplysiatoxin, 17-Debromo-, Leu-Lys-Ser, Plicatic Acid, Cholestane-3,7,12,25-Tetrol-3-Glucuronide, Lecanoric Acid, Myricanene A 5-[Arabinosyl-(1->6)-Glucoside], Clofibric Acid, Ala-Tyr-Trp, Ile-Leu-Leu, Tyr-Ile-Met, Asi-222, Fosinopril, 3-[(2-Methyl-3-Furanyl)Thio]-4-Heptanone, Ile-Ile-Ile, His-Ile-Gln, Trimethylcolchicinic Acid, Pro-Met-Val, Pectenotoxin 3, and Gly-Phe-Ile.

KEGG pathway annotation and enrichment analysis were performed on all significantly differential metabolites (those without relevant pathway information were not included in the analysis). The KEGG pathway annotation results (Figure 5F,G) showed that among all annotated pathways, “Metabolism” was the level 1 pathway with the highest number of annotated metabolites. For the up-regulated metabolites (Figure 5F), the primary level 2 pathways annotated included “Global and overview maps”, “Biosynthesis of other secondary metabolites”, and “Metabolism of terpenoids and polyketides”. For the down-regulated metabolites (Figure 5G), the most abundantly annotated level 2 pathway was “Global and overview maps”, followed by “Amino acid metabolism” and “Biosynthesis of other secondary metabolites”.

Further pathway enrichment analysis aimed to reveal statistically significant level 3 pathways. The results showed that upregulated differential metabolites were significantly enriched in the “Flavone and flavonol biosynthesis” pathway (*p*-adjust < 0.01) (Figure 5H), and the differential metabolites within this pathway included flavonoids such as Chrysoeriol and Laricitrin. Among the downregulated differential metabolites, no significantly enriched KEGG pathways were found (*p*-adjust > 0.05) (Figure 5I).

## 4. Discussion

### 4.1. Effects of Dietary Luteolin on the Growth Performance of Juvenile GIFT Tilapia

When evaluating the efficacy of feed additives, growth performance is one of the most closely monitored indicators in aquaculture. Previous studies have shown that adding flavonoid compounds to the feed can improve the physiological condition of animals, thereby enhancing feed utilization and growth performance [26,43,44]. In this study, the FCR was lowest in the LG300 group. Meanwhile, both the WGR and SGR initially increased and then decreased with increasing levels of dietary luteolin, peaking at a supplementation level of 300 mg/kg, indicating that the LG300 group exhibited the best growth performance. To further quantify the optimal inclusion level of luteolin, we fitted the WGR and SGR data using piecewise linear and quadratic polynomial regression models. The optimal inclusion levels determined by the two models were 279.72–303.39 mg/kg (based on WGR) and 274.14–302.74 mg/kg (based on SGR), both centered around 300 mg/kg. This further supported the finding that the LG300 group in this experiment represented the optimal dosage for promoting tilapia growth. We also found that within an appropriate supplementation range, luteolin could effectively improve the growth performance of fish, whereas excessive supplementation beyond this range might exert an inhibitory effect. A similar dose–response relationship was also observed in a study on red swamp crayfish, where the growth-promoting effect of luteolin peaked at 100 mg/kg and subsequently diminished with increasing dosage [26]. This suggests that the efficacy of luteolin is closely correlated with its supplementation level, exhibiting a characteristic of “promotion at low concentrations and inhibition at high concentrations.” This phenomenon has also been reported in studies on other flavonoids. For instance, the application of quercetin in snakehead fish (*Channa argus*) [44] and *Cyprinus carpio* [45] as well as the application of hesperidin in red swamp crayfish [18], all indicated that their growth-promoting effects were limited to a specific dosage range, with effects diminishing or being inhibited at high doses. Regarding the underlying mechanism of growth inhibition caused by high-dose luteolin, there is currently no consensus. One possible hypothesis is that flavonoid compounds may have “oxidation-promoting” properties at high concentrations [46]. These compounds can act as metal ion chelators, catalyzing the generation of reactive oxygen species (ROS) through Fenton-like reactions, or directly interfere with intracellular redox balance, leading to ROS accumulation exceeding the organism’s clearance threshold; this induces cell and tissue damage, thereby impairing intestinal absorption and overall metabolic processes.

### 4.2. Effects of Dietary Luteolin on Intestinal Antioxidant Enzyme Activities, Oxidative Stress, and Histology of Juvenile GIFT Tilapia

When reactive molecules such as ROS and reactive nitrogen species accumulate excessively or the body’s clearance capacity declines, oxidative stress is induced, leading to a series of physiological disorders and pathological responses. The body’s antioxidant enzyme system (e.g., SOD, CAT, GSH-Px) maintains redox homeostasis by scavenging free radicals, thereby ensuring normal bodily functions [47]. Studies in various fish species, including *Rhamdia quelen* [48], grass carp (*Ctenopharyngodon idella*) [49], common carp [50], snakehead fish [44], giant salamander (*Andrias davidianus*) [51], and gibel carp (*Carassius auratus* gibelio) [52], have confirmed that flavonoids can enhance the body’s antioxidant capacity.

Consistent with these reports, we also found that the LG150 and LG300 groups significantly increased the activity of intestinal T-SOD in juvenile GIFT tilapia, and the content of O_2_^−^ in the LG300 group was significantly reduced, indicating that appropriate amounts of luteolin can effectively improve the intestinal antioxidant status of fish. A study on rats suggested that luteolin could enhance the activity of downstream endogenous antioxidant enzymes, including SOD, by activating the Nrf2 signaling pathway, promoting the nuclear translocation of Nrf2, and upregulating the expression of HO-1 protein [53]. The increased T-SOD activity observed in this study may involve similar mechanisms; however, the specific signaling pathways by which luteolin plays a role in regulating the cellular response to oxidative stress in aquatic animals still require further exploration.

Furthermore, this study confirmed the dose-dependent effect of luteolin. When the inclusion level increased to 600 mg/kg, its promoting effect on T-SOD was no longer significant, a phenomenon similar to observations with puerarin [54] and quercetin [44] in other fish species. In the study of red swamp crayfish, it was also found that a high dose of luteolin (500 mg/kg) led to a decrease in antioxidant enzyme activity [26]. These results indicated that there exists an optimal concentration range for the action of luteolin. High doses might interfere with absorption and transport or exert a pro-oxidative effect, thereby limiting its positive impacts [46], and these findings were also consistent with the observed decrease in growth performance. Therefore, a supplementation level of 300 mg/kg was identified as the optimal dose in the feed for the present experiment, since the growth performance and intestinal antioxidant capacity of juvenile GIFT tilapia both reached their peak values at this dose. This also suggests that luteolin may promote fish growth by enhancing intestinal antioxidant capacity.

The integrity of the intestinal tissue structure serves as the morphological basis for nutrient digestion and absorption. Combined with the data above, we analyzed the intestinal histomorphology of the CG and LG300 groups. Different from the results showing that the flavonoids daidzein [54] and silymarin [44] could promote the optimization of intestinal fold morphology, no significant changes were observed in the height and number of intestinal folds in the LG300 group in this study. However, we also noticed that the thickness of the intestinal muscular layer in the LG300 group was significantly higher than that in the CG group. Thickening of the intestinal muscular layer enhances peristaltic capacity, accelerates chyme transit and optimizes the processes of digestion and absorption [55,56]. Therefore, it is speculated that luteolin may enhance intestinal peristalsis by promoting the proliferation and differentiation of intestinal smooth muscle cells, thereby improving the utilization efficiency of nutrients, and thus constitutes another important aspect of its growth-promoting mechanism.

### 4.3. Effects of Dietary Luteolin on the Intestinal Microbiota of Juvenile GIFT Tilapia

The intestinal microbiota is an important indicator reflecting intestinal health status [21], and flavonoids can exert beneficial effects through selective modulation of microbial structure [57]. In this study, the intestinal microbiota of the CG and LG300 groups was further analyzed. It was found that luteolin did not significantly alter the Alpha diversity of the tilapia intestinal microbiota, which is consistent with the experimental results of luteolin in meat rabbits [35]. However, this differs from the conclusions reported by [26] in red swamp crayfish, where luteolin increased the Chao1 and Observed species indices of the intestinal microbiota. The difference in results may stem from inherent distinctions in physiological structures, digestive tract microenvironments, and metabolic pathways for phytochemicals among different species. Nevertheless, Beta diversity analysis of the intestinal microbiota (PCoA plot) showed significant separation between samples from the CG and the LG300 group, indicating that luteolin could significantly alter the overall composition of the intestinal microbiota.

In the analysis of dominant phyla and genera, the selective regulatory effect of luteolin was clearer. We found that dietary supplementation with 300 mg/kg of luteolin reduced the relative abundance of Fusobacteriota and its subordinate genus *Cetobacterium* in the intestine. *Cetobacterium* is a core genus in the intestines of many freshwater fish, capable of producing vitamin B_12_ beneficial to the host [58,59]. However, some studies have shown that in cases of intestinal microbiota imbalance caused by high ammonia nitrogen stress [60] or by grass carp hemorrhage virus infection [61], the relative abundance of *Cetobacterium* tended to increase significantly. Therefore, in the context of these reports, the decreasing trend in the relative abundance of *Cetobacterium* in the LG300 group might reflect an adjustment of the gut microecological environment toward homeostasis, although this interpretation requires further verification. More importantly, the relative abundance of *Plesiomonas* in the LG300 group also decreased. *Plesiomonas* contains various opportunistic pathogens potentially pathogenic to both fish and humans, associated with gastroenteritis and sepsis [62]. This result indicated that the inhibitory effect of luteolin on these two genera might have positive significance for reducing the risk of pathogens in the gut. Concurrently, the relative abundance of Bacteroidota increased in the LG300 group. This phylum (particularly Bacteroidia) is rich in genes encoding carbohydrate-active enzymes, enabling the efficient degradation of complex carbohydrates indigestible by the host, such as dietary fiber and resistant starch. Short-chain fatty acids (SCFAs) produced by intestinal microbial degradation of these substances are an important energy source for intestinal epithelial cells, promoting their growth and differentiation and maintaining the mechanical barrier of the intestinal mucosa [63,64]. Furthermore, members of the Bacteroidota (such as *Bacteroides*) have been confirmed to participate in the deglycosylation process of flavonoids, a key initial step in the catabolism of flavonoids in vivo [65]. Therefore, the increase in Bacteroidota might enhance the microbiota’s carbohydrate degradation and SCFAs production capacity and might play a role in the biotransformation of luteolin.

LEfSe analysis further revealed profound changes in the microbial structure. The significant enrichment of *Romboutsia* in the LG300 group (undetected in the CG group) is noteworthy, as its abundance has been previously reported to positively correlate with dietary sugar levels in tilapia intestine and negatively correlate with *Cetobacterium* abundance [66,67]; the inverse relationship between *Romboutsia* and *Cetobacterium* in our study is consistent with those reports. Conversely, the relative abundance of *Bosea* was significantly higher in the CG group than in the LG300 group, in which it was undetected. *Bosea* is a Gram-negative aerobic bacterium widely distributed in environments such as water and soil, and it tends to proliferate more readily in intestinal environments with higher oxygen content [68]. In addition, this genus has been associated with microbiota dysbiosis under high plant-protein diets, and some of its strains have been proposed as potential environment-associated pathogens [69]. However, its specific role in the fish intestine remains unclear. Therefore, given that the LG300 group also achieved the best growth performance and intestinal antioxidant capacity, the reduction in *Bosea* in this group may be beneficial. Overall, dietary supplementation with 300 mg/kg luteolin optimized the intestinal microbiota of tilapia to some extent by increasing the relative abundance of potentially beneficial bacteria and inhibiting that of potentially pathogenic bacteria, thereby contributing to the overall improvement in growth performance and intestinal health.

### 4.4. Effects of Dietary Luteolin on the Intestinal Metabolism of Juvenile GIFT Tilapia

A growing body of research indicates that microbial-produced metabolites are key executors through which the intestinal microbiota exerts dietary effects on the host [63]. Luteolin, as a flavonoid, has been reported to have positive effects on the intestinal health of aquatic animals [26], but how it systematically regulates host metabolism in the intestine remains incompletely understood. This study employed an untargeted metabolomics approach for the first time to deeply investigate the effects of luteolin on the gut metabolic profile of juvenile GIFT tilapia. PLS-DA plot and OPLS-DA plot displayed distinct separation between the CG and the LG300 group, indicating that luteolin caused significant changes in gut metabolomic characteristics. The identified differential metabolites were mainly Organic acids and derivatives and Lipids and lipid-like molecules, indicating that luteolin intervened in organic acid and lipid metabolism within the intestine.

KEGG enrichment analysis showed that upregulated differential metabolites were significantly enriched in the “Flavone and flavonol biosynthesis” pathway, wherein the key metabolites were Chrysoeriol and Laricitrin. It is known that luteolin can be O-methylated into chrysoeriol in mammals [70], which also possesses biological activities such as anti-inflammatory and antibacterial effects [70,71]. This finding provided direct evidence that luteolin underwent Phase II metabolism within the tilapia intestine to exert biological activity (Phase II conjugation refers to processes such as methylation, glucuronidation, and sulfation). The digestive metabolism of flavonoids generally requires structural modification. For instance, after glycosides undergo hydrolysis or deglycosylation, phase II conjugation reactions occur in intestinal epithelial cells and the liver; subsequently, ring cleavage is catalyzed by bacterial or host enzymes to generate phenolic acid products [65,72].

Furthermore, the metabolite 5′-(3′,4′-Dihydroxyphenyl)-Gamma-Valerolactone Sulfate was significantly upregulated in the LG300 group. Its structure is similar to γ-valerolactone metabolites derived from the microbial transformation of catechins [73], suggesting that this substance might be one of the biomarkers for the microbial metabolism of luteolin. Hence, we propose that the beneficial effects of luteolin may be attributed to the synergistic action of its parent compound, together with its phase II metabolic products and microbial transformation derivatives.

It is worth mentioning that this study also identified changes in several other key metabolites. The content of Docosahexaenoylethanolamine (synaptamide) was significantly upregulated in the LG300 group; it is an endogenous fatty acid amide derived from docosahexaenoic acid (DHA), known to possess functions such as promoting synaptogenesis, neuroprotection, and anti-neuroinflammation [74,75]. This finding suggested that luteolin might participate in the regulation of the local intestinal neuro-metabolic axis by influencing the metabolism of DHA to generate the neuromodulatory mediator synaptamide. Of particular note is that luteolin also led to a significant downregulation of the biotoxins Aplysiatoxin, 17-Debromo- and Pectenotoxin 3. These toxins are synthesized by Cyanobacteria and *Dinophysis*; they can accumulate in aquaculture species and threaten animal health as well as food safety [76,77,78]. Luteolin has previously been shown to inhibit the growth of harmful algae [79]; therefore, its ability to reduce the content of these toxins in the intestine might constitute one of its important mechanisms for protecting intestinal health.

## 5. Conclusions

This study investigated the effects of dietary luteolin supplementation on the growth performance and intestinal health of juvenile GIFT tilapia. The results showed that dietary supplementation with 300 mg/kg luteolin could significantly improve fish growth performance, enhance intestinal antioxidant enzyme activity, and improve intestinal morphological structure. Broken-line and quadratic polynomial regression analyses determined that the optimal supplementation levels based on weight gain rate and specific growth rate were 279.72–303.39 mg/kg and 274.14–302.74 mg/kg, respectively. Furthermore, at the supplementation level of 300 mg/kg, luteolin increased the relative abundance of potential probiotics such as Bacteroidota and *Romboutsia*, while reducing the relative abundance of potential pathogens such as *Plesiomonas* and *Bosea*. Metabolomic analysis showed that luteolin could significantly regulate the intestinal “Flavone and flavonol biosynthesis” pathway, promote the production of beneficial bioactive metabolites and reduce toxic metabolites. In conclusion, our experimental results demonstrated that luteolin could improve the intestinal health and growth performance of juvenile GIFT tilapia. Moreover, this study filled a research gap regarding the application of luteolin in tilapia and provided a theoretical basis for its scientific application in aquaculture.

Although experimental approaches in this study already provided novel and robust evidence for the effects of luteolin on intestinal health, future studies should add more analysis (for example, targeted qPCR of key genes, transcriptomic, and proteomics) to fully decipher the molecular regulatory network underlying the beneficial effects of luteolin.

## Figures and Tables

**Table 1:** Formulation and nutrient levels of the basal diet (dry matter).

Ingredients (%)		Nutrient Levels (%) ^c^	
Fish meal	8.5	Crude protein	32.90
Soybean meal	40	Crude lipid	3.81
Rape seed meal	20	Crude fiber	16.90
Wheat middlings	13	Crude ash	6.40
Calcium phosphate	1.5	Nitrogen-free extract	39.99
Choline Chloride	1.5	Gross energy (kJ/g)	19.05
Vitamin premix ^a^	0.5		
Mineral premix ^b^	0.5		
Microcrystalline cellulose	9		
NaCl	0.5		
Soybean oil	2		
Carboxymethyl cellulose	3		
Total	100		

Notes: ^a^ The vitamin premix and mineral premix were purchased from Haibaolu Feeds Co., Ltd. (Nanning, China). The vitamin premix provided the following per kilogram of premix: thiamine (VB_1_) 10 mg, riboflavin (VB_2_) 8 mg, pyridoxine hydrochloride (VB_6_) 10 mg, cyanocobalamin (VB_12_) 0.2 mg, menadione (VK_3_) 10 mg, inositol 100 mg, calcium pantothenate 20 mg, nicotinic acid 50 mg, folic acid 2 mg, biotin 2 mg, vitamin A 400 mg, vitamin D 5 mg, vitamin E 100 mg, ethoxyquin 150 mg, and wheat middlings (carrier) 132.8 mg. ^b^ The mineral premix provided the following per kilogram of premix: potassium chloride 200 mg, potassium iodide 60 mg, cobalt sulfate (1%) 100 mg, copper sulfate 24 mg, ferrous sulfate 400 mg, zinc sulfate 174 mg, manganese sulfate 78 mg, magnesium sulfate 800 mg, sodium selenite (1%) 50 mg, and zeolite powder (carrier) 3114 mg. ^c^ All nutrient levels were calculated using the VF123 feed formulation software (version 2016; Beijing Jinmu Times Technology Co., Ltd., Beijing, China).

**Table 2:** Effects of luteolin-supplemented diet on the growth performance of juvenile GIFT tilapia.

Item	Group					
	CG	LG50	LG150	LG300	LG600	*p*
IBW (g)	8.13 ± 0.03	8.11 ± 0.05	8.12 ± 0.04	8.09 ± 0.05	8.14 ± 0.05	0.611
FBW (g)	70.51 ± 2.88	73.64 ± 5.84	77.06 ± 3.72	81.64 ± 6.45	70.71 ± 1.33	0.084
WGR (%)	767.71 ± 35.85 ^b^	807.90 ± 70.24 ^ab^	848.68 ± 7.30 ^ab^	909.54 ± 77.84 ^a^	768.40 ± 21.15 ^b^	0.029
SGR (%/d)	3.93 ± 0.08 ^b^	4.01 ± 0.14 ^ab^	4.09 ± 0.01 ^ab^	4.20 ± 0.1 4 ^a^	3.93 ± 0.04 ^b^	0.029
FR (%/d)	4.09 ± 0.35	3.88 ± 0.08	3.82 ± 0.05	3.64 ± 0.10	3.83 ± 0.13	0.212
FCR	1.45 ± 0.16	1.36 ± 0.03	1.33 ± 0.05	1.24 ± 0.04	1.34 ± 0.04	0.082

Notes: Data are expressed as mean ± SD (*n* = 3 per group, total *n* = 15). Within the same row, values that do not share same superscript letter are significantly different, whereas values sharing same letter or with no letter do not differ significantly (one-way ANOVA followed by post hoc test; *p* < 0.05 indicates significant difference). CG: control group; LG50, LG150, LG300 and LG600: groups supplemented with 50, 150, 300 and 600 mg/kg luteolin in the basal diet, respectively. IBW: initial body weight; FBW: final body weight; WGR: weight gain rate; SGR: specific growth rate; FR: feeding rate; FCR: feed conversion ratio.

**Table 3:** Comparison of intestinal morphology of juvenile GIFT tilapia between the CG and LG300 groups.

Group	Fold Height (μm)	Fold Number	Thickness of Muscular Layer (μm)
CG	190.31 ± 33.25	15.33 ± 3.21	31.29 ± 4.94
LG300	214.96 ± 22	15 ± 3.46	45.93 ± 6.92
*p*	0.345	0.909	0.041

Notes: Data are expressed as mean ± SD (*n* = 3 per group, total *n* = 6). *p* values were obtained from an independent-samples *t*-test comparing the CG and LG300 groups; *p* < 0.05 indicates significant difference. CG: control group; LG300: group supplemented with 300 mg/kg luteolin in the basal diet.

**Table 4:** Number of identified and annotated intestinal metabolites of juvenile GIFT tilapia from the CG and LG300 groups.

Ionization Mode	Number of Identified Metabolites	Number of Metabolites Annotated in HMDB	Number of Metabolites Annotated in KEGG
Positive Mode	1923	989	413
Negative Mode	2427	1679	639

**Table 5:** Top 20 significantly up- and down-regulated metabolites in the LG300 group compared with the CG group.

Metabolite	*p*-Adjust	VIP	FC
**Up-regulated**			
5′-(3′,4′-Dihydroxyphenyl)-Gamma-Valerolactone Sulfate	0.0060	2.8932	2.1362
Chrysoeriol	0.0078	1.7525	1.3120
Propranolol Glucuronide	0.0117	1.8431	1.2902
Diosmetin	0.0157	1.5064	1.1921
Meosuc-Aapa-Cmk	0.0191	1.9926	1.3443
4-Hydroxy-5-(Phenyl)-Valeric Acid-O-Sulphate	0.0233	2.6372	2.0182
Quinoneimine	0.0268	1.8468	1.2831
Selpercatinib	0.0294	1.7833	1.2486
Laricitrin	0.0339	1.7620	1.3281
2-C-Methyl-D-Erythritol 2,4-Cyclodiphosphate	0.0345	2.0637	1.4230
Docosahexaenoylethanolamine	0.0350	1.7099	1.2406
Kaempferol 3-Glucuronoside	0.0377	1.7291	1.4257
Pe(5-Iso Pgf2Vi/22:6(4Z,7Z,10Z,13Z,16Z,19Z))	0.0406	1.5753	1.2175
3-Methyladipic Acid	0.0407	1.6344	1.2001
Glu-Phe-Tyr	0.0409	1.6949	1.2691
Elarofiban	0.0410	1.7211	1.2244
Glu-Ile-Phe-Lys	0.0419	1.5589	1.2181
Cyclo[Dl-Pro-Dl-Pro-Dl-Val-Dl-Tyr-Dl-Tyr]	0.0424	1.5209	1.1855
Trinexapac	0.0430	1.7163	1.4642
Ginsenoside A2	0.0457	1.7080	1.2877
**Down-regulated**			
Gly-Glu-Arg	0.0174	2.0927	0.7356
Aplysiatoxin, 17-Debromo-	0.0188	2.0286	0.7635
Leu-Lys-Ser	0.0205	2.0804	0.6769
Plicatic Acid	0.0209	1.9452	0.7043
Cholestane-3,7,12,25-Tetrol-3-Glucuronide	0.0294	2.0714	0.7429
Lecanoric Acid	0.0296	2.6861	0.4690
Myricanene A 5-[Arabinosyl-(1→6)-Glucoside]	0.0300	2.4601	0.6019
Clofibric Acid	0.0300	1.9290	0.7142
Ala-Tyr-Trp	0.0313	2.1082	0.7090
Ile-Leu-Leu	0.0342	1.9497	0.7770
Tyr-Ile-Met	0.0343	1.8121	0.8041
Asi-222	0.0343	1.5780	0.8289
Fosinopril	0.0345	1.9170	0.7979
3-[(2-Methyl-3-Furanyl)Thio]-4-Heptanone	0.0345	1.5825	0.8387
Ile-Ile-Ile	0.0350	1.6868	0.8231
His-Ile-Gln	0.0376	2.3136	0.6581
Trimethylcolchicinic Acid	0.0377	2.5471	0.6081
Pro-Met-Val	0.0377	2.4022	0.5684
Pectenotoxin 3	0.0377	1.7788	0.8136
Gly-Phe-Ile	0.0377	1.6754	0.8469

Notes: *n* = 6 per group, total *n* = 12. VIP: variable importance in projection; FC: fold change (LG300/CG); *p*-adjust: Benjamini–Hochberg (BH) adjusted *p*-value; *p*-adjust < 0.05 indicates significant difference; CG: control group; LG300: group supplemented with 300 mg/kg luteolin in the basal diet.

## Data Availability

The original contributions presented in this study are included in the article. Further inquiries can be directed to the corresponding author.
